# Molecular Cloning and Functional Characterization of Δ9 Fatty Acyl Desaturase in *Scylla paramamosain*: Insights into Its Regulatory Roles Under Salinity Challenge, Dietary Fatty Acid Alteration and Starvation Stress

**DOI:** 10.3390/life16081372

**Published:** 2026-08-20

**Authors:** Zhideng Lin, Zhaohan Sun, Yunwang Chen, Chengmei Li, Rong Chen, Kunhuang Han, Weiqing Huang

**Affiliations:** 1 College of Marine Sciences, Ningde Normal University, Ningde 352100, China; 2 Ningde Yiye Marine Industry Development Co., Ltd., Ningde 352100, China

**Keywords:** Δ9 fatty acyl desaturase, cloning, regulatory mechanisms, *Scylla paramamosain*, environment stress

## Abstract

Δ9 fatty acyl desaturase (Δ9 FAD) exerts important regulatory effects on fatty acid metabolism and membrane fluidity. However, its existence and the effects of nutritional regulation and ambient salinity on its expression are currently unknown in mud crab (*Scylla paramamosain*). In this study, the full cDNA sequence of *Δ9 fad* was isolated from the hepatopancreas of mud crab, which consists of 1468 bp and encodes a polypeptide of 347 amino acids. Sequence analysis revealed that the protein possessed typical characteristics of Δ9 FAD including three histidine-rich domains (HRLWSH, HRVHH and HNYHH), four transmembrane regions and three conserved motifs (FFSHMGW, QRKYY and PWDY). The results of tissue distribution indicated that the mud crab’s *Δ9 fad* expression was enriched in the hepatopancreas, with relatively lower levels found in the thoracic ganglia and cranial ganglia. *Δ9 fad* expression in the hepatopancreas was significantly influenced by dietary fatty acid profiles, and the replacement of fish oil with soybean oil markedly downregulated the transcriptional levels of hepatopancreatic *Δ9 fad*. Furthermore, the transcriptional levels of *Δ9 fad* were also significantly affected by ambient salinity. Under chronic salinity stress, the 7‰, 12‰, 17‰ and 22‰ salinity groups showed higher transcriptional levels of *Δ9 fad* in the hepatopancreas than the 27‰ salinity group, and the 7‰ salinity group significantly differed from the 27‰ salinity group. Under the acute salinity stress condition, the *Δ9 fad* transcriptional levels of muscle in the 14‰ and 4‰ salinity groups were higher than those in the 24‰ salinity group at 6 h and 12 h. By contrast, *Δ9 fad* in the hepatopancreas exhibited a slow response in reaction to low salinity stress, and only the 4‰ salinity group at 96 h had a higher value than the 24‰ salinity group. In addition, as for the starvation stress experiment, *Δ9 fad* expression in the starvation group was dramatically reduced when compared with the feeding group. These findings improve our understanding of the physiological roles of *Δ9 fad* and its regulatory mechanisms in crustaceans.

## 1. Introduction

Fatty acyl desaturases regulate the unsaturation of fatty acids by introducing a double bond at specific positions of the acyl-coenzyme A (CoA) substrate and can be classified based on the length of fatty acyl chains and the position of the introduced double bond [1]. Δ9 fatty acyl desaturase (Δ9 FAD), a kind of membrane-bound desaturase located in the endoplasmic reticulum membrane, primarily mediates the insertion of a double bond between the C9 and C10 positions in stearoyl-CoA (C18:0) and palmitoyl-CoA (C16:0) from saturated fatty acids (SFAs) [2,3]. Acting as a rate-limiting enzyme in lipogenesis, Δ9 FAD not only participates in the synthesis of monounsaturated fatty acids (MUFAs) but also maintains membrane fluidity and energy balance [3,4]. Structural studies showed that Δ9 FAD possesses four transmembrane regions consisting of a cytosol region, carboxy terminus, amino terminus as well as a histidine region, and a histidine-rich region, which contains eight catalytically essential histidines, functioning as potential iron atom ligands [5]. To date, many studies have been performed to investigate the evolutionary history, structural features, tissue distribution, physiological functions and regulatory mechanisms of *Δ9 fad* in mammals [3,4,5,6,7]. In comparison, the physiological roles of *Δ9 fad* remain poorly characterized in crustaceans, and studies have merely covered *Eriocheir sinensis* [8] and *Cherax quadricarinatus* [9]. Specifically, beyond fundamental gene characterization, these two studies investigated distinct regulatory triggers governing *Δ9 fad* transcription. One characterized the modulatory effects of dietary lipid sources on hepatopancreatic gene expression in *E. sinensis* [8], whereas the other demonstrated the transcriptional and enzymatic upregulation of this desaturase in *C. quadricarinatus* subjected to chronic cold stress, highlighting its functional contribution to cold-temperature adaptation [9].

Many studies in mammals suggested that Δ9 FAD was intimately related to metabolic diseases including inflammation [10,11], atherosclerosis [6], insulin resistance [12], heart disease [13] and cancer [7] by altering the ratio of MUFAs and SFAs. *Δ9 fad* gene expression was notably influenced by nutritional factors [8], temperature variation [9], metal ions [14], alcohols [15] and diverse hormones [16], and it was positively adjusted by transcription factors like liver X receptor (LXR), carbohydrate response element-binding protein (CHREB) and sterol-regulatory element-binding protein-1c (SREBP-1c) [2]. For instance, dietary carbohydrates could activate SREBP-1c and LXR through stimulating insulin secretion, finally promoting *Δ9 fad* gene expression [17]. In addition to carbohydrates, diets supplemented with polyunsaturated fatty acids (PUFAs) markedly downregulated the expression of *Δ9 fad* by inhibiting *srebp-1c* transcription [3,4]. In addition, *Δ9 fad* plays a crucial part in coping with cold stress through promoting MUFA synthesis and increasing the degree of unsaturation in phosphoglycerides to alleviate cold-triggered membrane rigidification and recover membrane fluidity [9,18,19,20,21]. In carp liver, cold treatment triggered an 8- to 10-fold elevation in the specific activity of microsomal Δ9 FAD [18]. A similar result was also detected in *C. quadricarinatus*, in which it was observed that cold stress markedly increased *Δ9 fad* transcript levels and its enzymatic activity [9]. These results indicated that *Δ9 fad* played a crucial role in coping with temperature stress. Apart from temperature variation, salinity and starvation are also common stress factors, while how *Δ9 fad* expression responds to starvation and ambient salinity stress remains poorly documented in aquatic animals.

The mud crab, *Scylla paramamosain*, is a commercially significant aquaculture species with broad cultivation areas covering southern China and multiple Indo-Pacific countries. The mud crab has become a desired food for people due to its good taste, rich nutrition and fleshiness. Mud crab production in China amounted to 163,512 tons in 2025 owing to advances in culture techniques, artificial propagation and larval rearing [22,23]. In this study, we investigated the molecular characterization of *Δ9 fad* and its expression in response to dietary fatty acids, salinity stress and starvation stress. These findings will advance our understanding of the functions of *Δ9 fad* and its underlying regulatory mechanisms in crustaceans.

## 2. Materials and Methods

### 2.1. Nutritional and Stress Experiments

Six isonitrogenous and isolipidic experimental diets (9.5% crude lipid and 45% crude protein) were formulated with the graded soybean oil substitution of fish oil. Specifically, the six diets were named SO-0, SO-20, SO-40, SO-60, SO-80 and SO-100, representing soybean oil replaced by fish oil at 0, 20, 40, 60, 80 and 100% respectively. The feeding trial lasted for eight weeks, and juvenile *S. paramamosain* with an initial body weight of 20.19 ± 0.82 g were used in this experiment on the replacement of fish oil with vegetable oil. Hepatopancreas samples were obtained from nine crabs per treatment for subsequent gene expression assays (*n* = 9). Detailed information regarding the fish oil substitution experiment has been described in our previous studies [24,25], and diet formulation and its fatty acid profile are presented in Tables S1 and S2, respectively. Four treatment groups (7‰, 12‰, 17‰, and 22‰ salinity) and one control group (27‰ salinity) were set up for the chronic low-salinity stress experiment, whereas the starvation stress experiment comprised a starved treatment group and a fed control group. The chronic low-salinity stress trial was conducted over a 21-day period, and 90 crabs with an initial average weight of 62.90 ± 1.98 g were assigned to five treatments with three biological replicates and six crabs per replicate. For the starvation stress trial, crabs with an initial average weight of 64.51 ± 0.83 g were used, and the experiment lasted for 28 days. Upon the termination of each trial, all crabs were subjected to a 24 h fasting period prior to dissection. Hepatopancreas and muscle samples were obtained from six crabs per treatment for subsequent gene expression assays (*n* = 6). Collected hepatopancreas and muscle tissues were immediately snap-frozen in liquid nitrogen and preserved at −80 °C pending subsequent analyses. Detailed information on the experimental layout, culture conditions and sampling procedures of these two trials has been reported previously [26].

The acute low-salinity stress assay was implemented using the aquaculture facility at Ningde Normal University. Well-conditioned mud crab acquired from a commercial farm located in Sandu Bay (Ningde, Fujian, China) underwent temporary rearing for acclimation prior to experimental manipulation. Three salinity regimes were established in this trial, consisting of one control group maintained at 24‰ and two treatment groups exposed to 14‰ and 4‰. A total of 72 active mud crabs with an average body weight of 68.66 ± 1.38 g were randomly distributed into 12 polypropylene tanks (Zhongkehai, Qingdao, China). All experimental crabs were maintained at the intermolt stage, and males and females were included at an equal 1:1 ratio. The experiment comprised three treatments with four biological replicates per group, and six crabs were stocked in each replicate. Throughout the experimental period, experimental crabs were supplied with the local razor clam *Sinonovacula constricta*, and seawater renewal was performed twice each day. One individual crab was sampled per biological replicate at each time point (0–96 h). Tissue samples including those of the gill, muscle and hepatopancreas were dissected at 0, 6, 12, 24, 48 and 96 h post salinity challenge. Gill, muscle and hepatopancreas samples were obtained from four crabs per treatment for subsequent gene expression assays (*n* = 4). All collected tissues were snap-frozen in liquid nitrogen immediately after dissection and preserved at −80 °C until subsequent RNA isolation.

### 2.2. RNA Extraction, cDNA Reverse Transcription and Gene Cloning

Fresh hepatopancreatic tissues were collected, and total RNA was extracted with Trizol reagent (Invitrogen, Carlsbad, CA, USA) to prepare cDNA templates for subsequent gene cloning. Following the evaluation of the integrity and purity of total RNA, qualified RNA samples were subjected to reverse transcription to generate first-strand cDNA. The synthesis reaction was carried out employing the SMART RACE cDNA Amplification kit (Clontech, Mountain View, CA, USA) following the manufacturer’s instructions. The resulting cDNA was preserved at −20 °C for subsequent experimental analyses.

Partial cDNA fragments encoding the mud crab *Δ9 fad* gene were retrieved from the transcriptomic dataset generated in our prior research. Gene-specific primers were designed relying on this partial sequence to amplify the 3′- and 5′-untranslated regions (UTRs) of *Δ9 fad*, and all cloning primers are listed in Table 1. A combination of touchdown PCR as the primary amplification and nested PCR as the secondary amplification was adopted, and the 3′- and 5′-UTRs were separately amplified via the rapid amplification of cDNA ends (RACE). Detailed reaction systems and thermal cycling parameters for the touchdown and nested PCRs were described in our earlier reports [24,25]. The full-length *Δ9 fad* sequence was further verified using a high-fidelity DNA polymerase. The verification PCR system with a total volume of 25 μL consisted of 9.5 μL PCR-grade water, 1 μL C-Δ9 Fad forward primer, 1 μL C-Δ9 Fad reverse primer, 12.5 μL 2 × Pfu PCR mix (Tiangen, Beijing, China), and 1 μL cDNA template. The thermal profile was set as follows: initial denaturation at 95 °C for 3 min; 35 amplification cycles comprising denaturation at 95 °C for 30 s, annealing at 58 °C for 30 s and extension at 72 °C for 90 s; followed by a final extension at 72 °C lasting 5 min. Amplified target products were separated via 1% agarose gel electrophoresis, and fragment purification was conducted using the TIANgel MIDI Purification Kit (Tiangen, Beijing, China). Recombinant plasmids were subsequently sent to BGI (Shenzhen, China) for commercial sequencing.

### 2.3. Sequence Analysis and Phylogenetic Tree Construction

Sequence similarity and identity were determined via BLAST from the National Center for Biotechnology Information (http://www.ncbi.nlm.nih.gov/, accessed on 17 August 2026). DNAMAN was applied to align the Δ9 FAD protein sequences derived from mud crab and additional crustaceans. The transmembrane topology of mud crab Δ9 FAD was predicted using the TMHMM 2.0 online server (https://services.healthtech.dtu.dk/services/TMHMM-2.0/) (accessed on 15 April 2025). Multiple sequence alignment was performed using ClustalW, and a neighbor joining (NJ) phylogenetic tree was constructed in MEGA 7.0 (Mega Software, Tempe, AZ, USA) with the p-distance substitution model. Bootstrap analysis with 10,000 replicates was applied to evaluate the reliability of branch topologies within the generated phylogenetic tree.

### 2.4. Quantitative Real-Time PCR (qRT-PCR)

Six mud crabs (female: male = 1:1, *n* = 6) with an average body weight of 103.60 ± 6.20 g were sampled to detect the transcriptional profiles of *Δ9 fad* across various tissues to determine the tissue distribution by using the qRT-PCR method. This approach was also used to examine transcriptional changes in *Δ9 fad* under low-salinity challenge, dietary fatty acid treatment and starvation stress. All primer sequences applied for qRT-PCR are listed in Table 1. The qRT-PCR mixture and thermal cycling parameters for detecting tissue distribution and gene responses to dietary fatty acids have been described in our previous work [24,25]. For starvation and salinity stress trials, the total RNA of tissue samples was extracted using TRNzol Universal Reagent (Tiangen, Beijing, China). Subsequently, 1 μg of good-quality total RNA was reverse-transcribed into cDNA employing the PrimeScript^®^ RT reagent Kit with gDNA Eraser (Takara, Dalian, China), and the obtained cDNA was diluted fourfold with ultrapure water for subsequent analysis. The qRT-PCR thermal program consisted of an initial denaturation step at 95 °C for 30 s, followed by 40 amplification cycles (95 °C for 10 s; 60 °C for 30 s). A melting curve analysis (95 °C for 15 s, 60 °C for 60 s, 95 °C for 15 s) was performed to verify the specificity of amplified products. qRT-PCR amplifications were conducted in a 20 μL reaction system, which contained 1.0 μL diluted cDNA template, 8.2 μL sterile distilled water, 10 μL of 2 × ChamQ Universal SYBR qPCR Master Mix (Vazyme, Nanjing, China) and 0.4 μL of each primer (10 μM). Serial fivefold diluted cDNA samples were utilized to generate standard curves. The amplification efficiency of each primer pair was calculated according to the equation E = 10^(−1/Slope)^ − 1, and values ranging from 95% to 105% were obtained in this study. Relative transcript abundance was calculated based on the 2^−ΔΔCt^ algorithm [27].

### 2.5. Statistical Analysis

All statistical analyses were performed using SPSS 20.0. All data are expressed as means ± standard error of the mean (SEM). An independent-samples *t*-test was utilized to examine statistical differences in the starvation stress trial. For the vegetable oil replacement trial, low-salinity challenge experiment and tissue distribution, a one-way analysis of variance (ANOVA) coupled with Duncan’s multiple range test was adopted to detect intergroup variations. In addition, prior to statistical analysis, the Shapiro–Wilk test was used to verify the normality of data, and Levene’s test was adopted to examine homogeneity of variance. All datasets satisfied the assumptions for the ANOVA in the present study. Differences were regarded as statistically significant when *p* < 0.05.

## 3. Results

### 3.1. Sequence Structural Analysis

**Figure 1:**
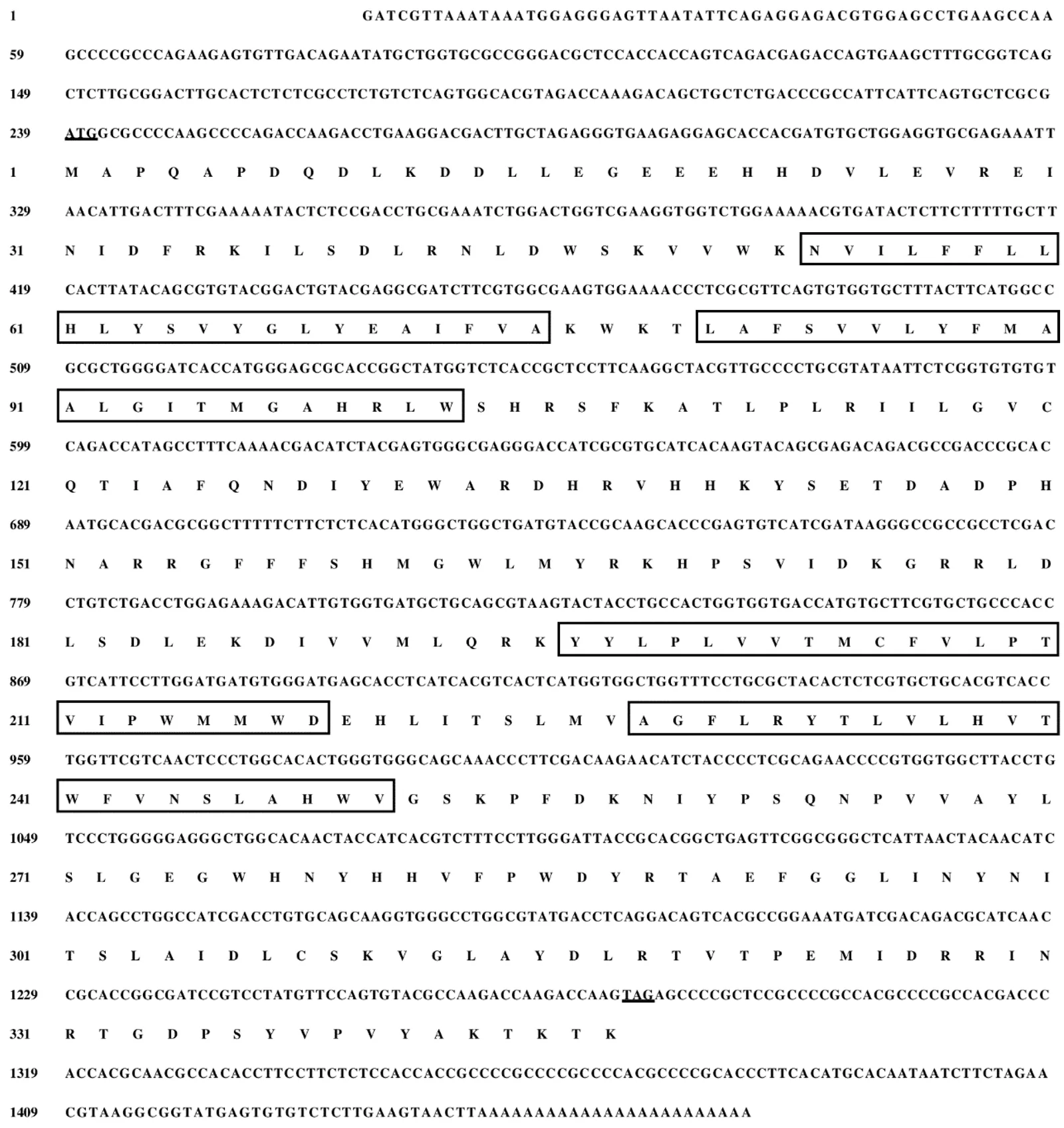
The full-length cDNA nucleotide sequence and corresponding encoded amino acid sequence of *Scylla paramamosain* Δ9 fatty acyl desaturase. The 1468 bp completed cDNA includes a 5′-untranslated region (UTR, positions 1–238), an open reading frame (ORF, positions 239–1282) and a 3′-UTR (positions 1283–1468). Underlined sequences indicate the start codon (ATG) and termination codon (TAG). Predicted membrane-spanning domains are boxed. GenBank accession number MF124319.

**Figure 2:**
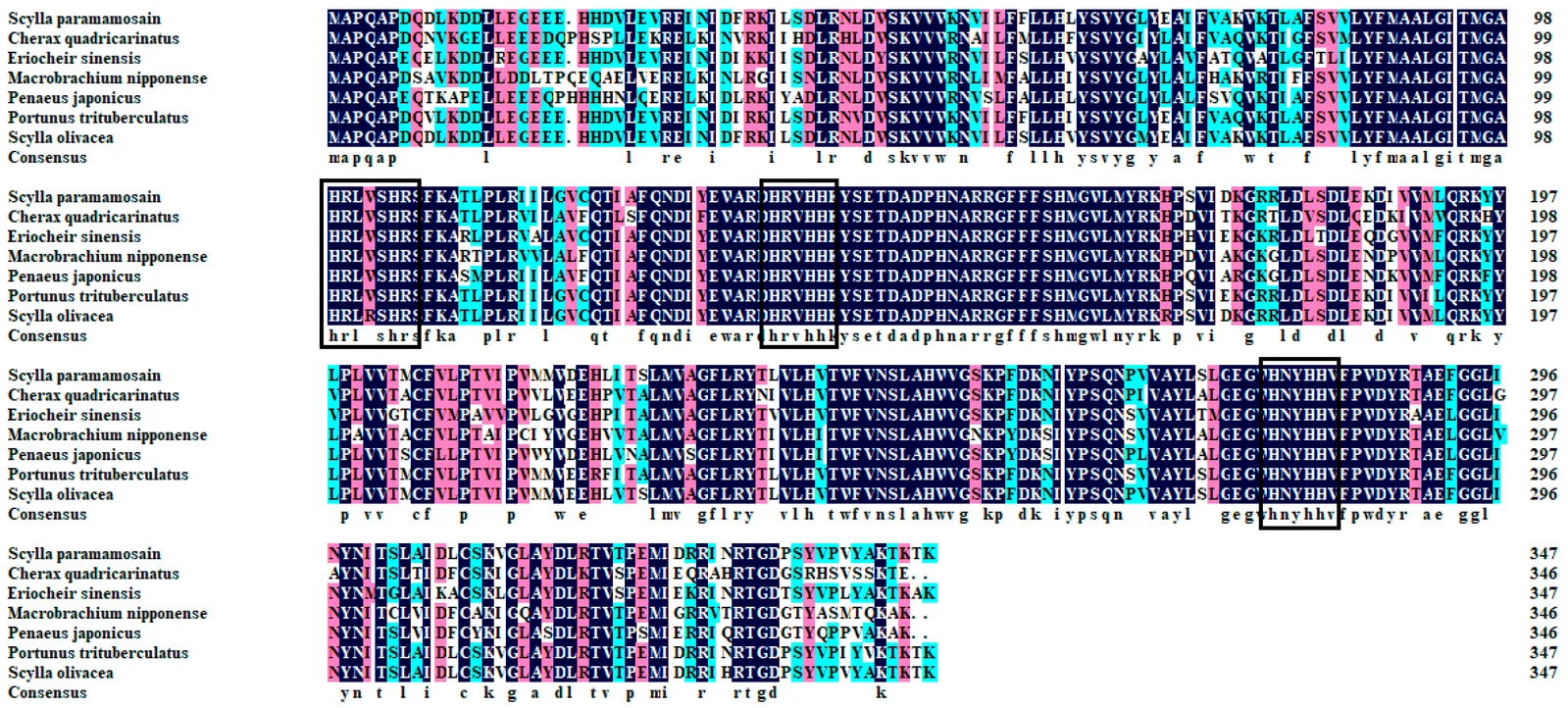
The multiple sequence alignments of Δ9 fatty acyl desaturase proteins from *Scylla paramamosain* with other crustaceans. Sequence similarity shading was performed using a 50% threshold. Black shading denotes identical residues, and pink and cyan shading represent residues with >75% and 50–75% similarity, respectively. Three conserved histidine rich motifs are boxed. The sequences used for comparison are as follows: *Scylla paramamosain* (ASU87504), *Cherax quadricarinatus* (XP_053644453), *Eriocheir sinensis* (XP_050736646), *Macrobrachium nipponense* (AMQ48727), *Penaeus japonicas* (XP_042859426), *Portunus trituberculatus* (XP_045124725) and *Scylla olivacea* (QKG32713).

RACE technology was utilized to obtain the full-length *Δ9 fad* cDNA sequence from the hepatopancreas of mud crab, and the sequence was deposited in GenBank under accession number MF124319. The nucleotide and deduced amino acid of *Δ9 fad* are presented in Figure 1. The total length of this cDNA is 1468 bp, including a 238 bp 5′-UTR, a 186 bp 3′-UTR with a poly(A) tail, and a 1044 bp open reading frame (ORF) responsible for encoding a polypeptide of 347 amino acids. Sequence analysis revealed that the protein possessed typical characteristics of Δ9 FAD including three histidine-rich domains (HRLWSH, HRVHH and HNYHH), four transmembrane regions and three conserved motifs (FFSHMGW, QRKYY and PWDY) (Figure 2).

### 3.2. Homology and Phylogenetic Tree Analysis

**Figure 3:**
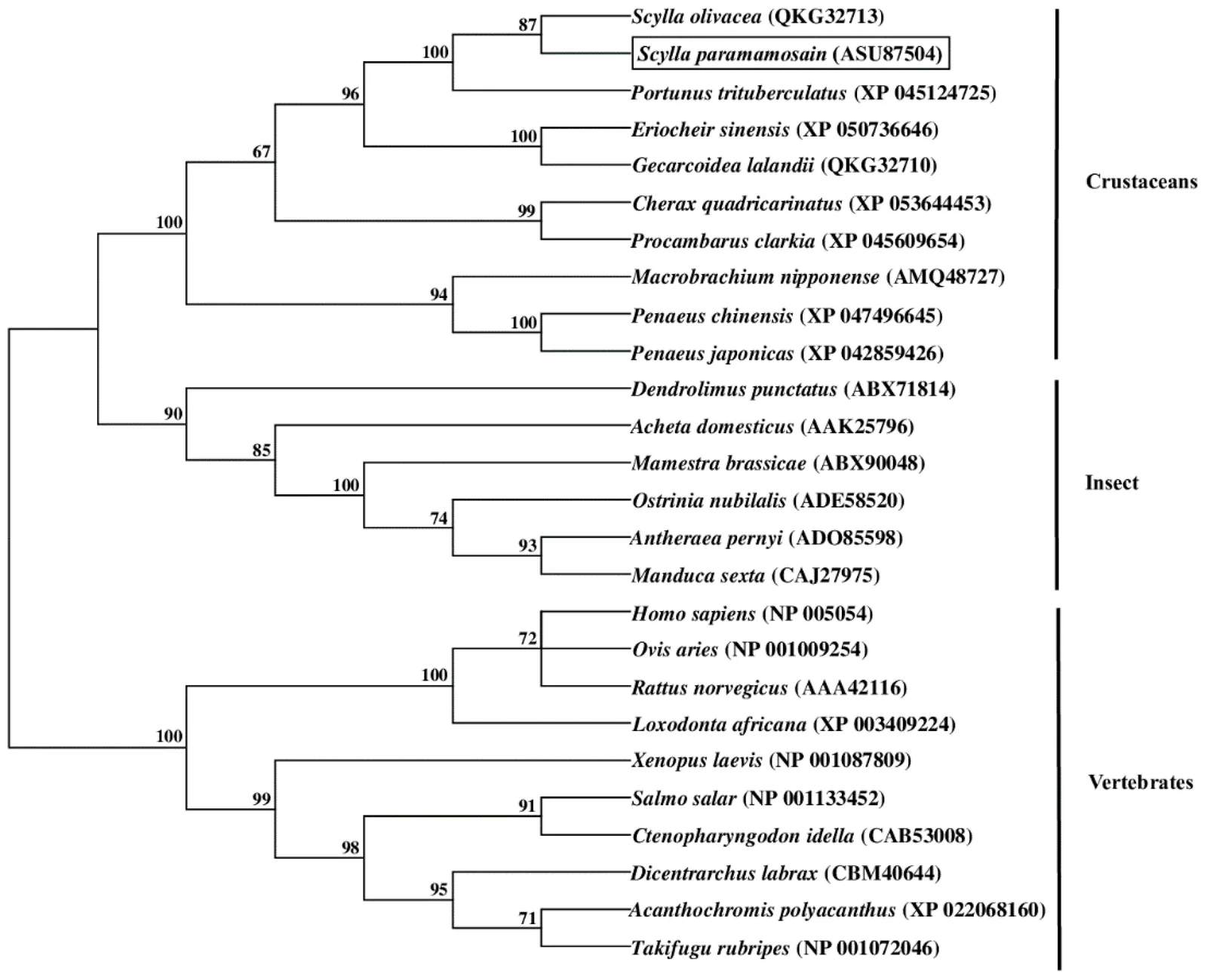
A phylogenetic tree of Δ9 fatty acyl desaturase amino acid sequences from representative invertebrates and vertebrates constructed via the neighbor joining method. Phylogenetic inference was carried out in MEGA 7.0. Horizontal branch lengths reflect the amino acid substitution at each amino acid site. Nodal numbers indicate the bootstrap support values calculated from 1000 replicate analyses.

The predicted protein of mud crab Δ9 FAD shares 68% to 98% identity scores with 10 reported Δ9 FADs from other crustaceans, *Scylla olivacea* (98%), *Portunus trituberculatus* (96%), *Gecarcoidea lalandii* (86%), *E. sinensis* (83%), *Penaeus japonicas* (80%), *Penaeus monodon* (80%), *Penaeus chinensis* (80%), *C. quadricarinatus* (78%), *Macrobrachium nipponense* (76%) and *Procambarus clarkia* (68%) (Table 2). Phylogenetic tree analysis indicated that the Δ9 FAD from mud crab grouped with crustacean orthologs, forming a distinct clade separate from insects and vertebrates. Moreover, the mud crab’s Δ9 FAD exhibited the closest evolutionary relationship with the counterpart from *S. olivacea*, followed by *P. trituberculatus* (Figure 3).

### 3.3. Tissue Distribution

**Figure 4:**
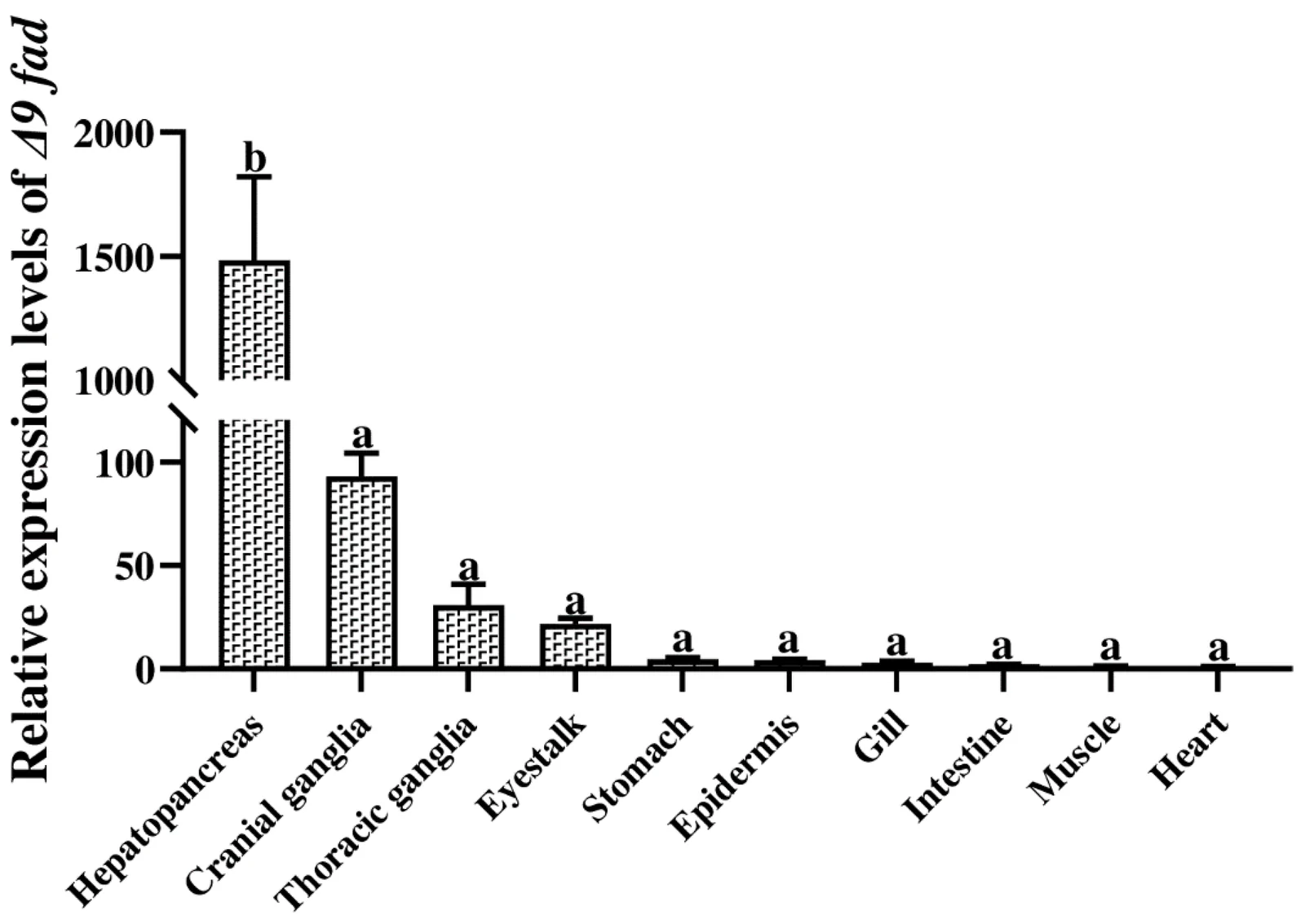
Differential mRNA abundance of *Scylla paramamosain* Δ9 fatty acyl desaturase in various tissues. Values are presented as mean ± standard error of mean (SEM, *n* = 6). Different letters indicate statistically significant differences (*p* < 0.05) among tissues.

*Δ9 fad* transcripts were present in all tested mud crab tissues, but expression levels differed markedly among tissues. *Δ9 fad* was highly expressed in the hepatopancreas when compared with other tissues (*p* < 0.05). Comparatively high transcript abundance was also found in cranial ganglia and thoracic ganglia, albeit with no significant differences. Low levels of expression were detected in the eyestalk, stomach, epidermis, gill, intestine, muscle and heart (Figure 4).

### 3.4. Transcriptional Levels of Δ9 fad in Response to Vegetable Oil Replacement Trial

**Figure 5:**
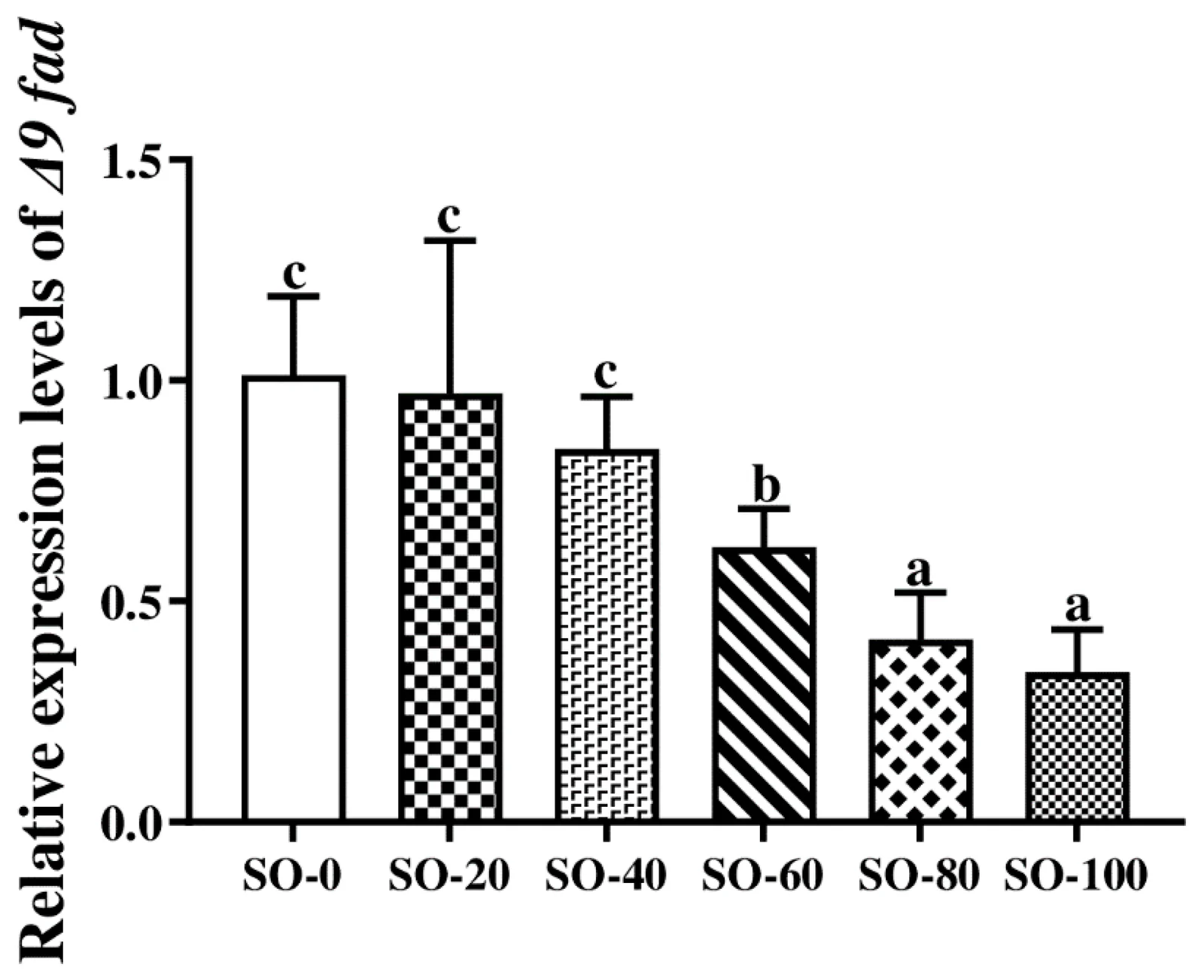
The differential mRNA abundance of Δ9 fatty acyl desaturase in the hepatopancreas of *Scylla paramamosain* fed with different diets. Values are expressed as the mean ± standard error of the mean (*n* = 9). Significant differences are denoted by distinct superscripts (*p* < 0.05). SO-0 denotes the group with the exclusive dietary lipid source, and numbers after SO correspond to the proportion of fish oil replaced by soybean oil.

Changes in dietary fatty acid composition significantly modulated *Δ9 fad* expression in the hepatopancreas. Elevated soybean oil replacement for dietary fish oil resulted in a gradual reduction in *Δ9 fad* transcript levels. Specifically, compared with the fish oil group (control group, SO-0), the mRNA level of *Δ9 fad* was significantly downregulated by about 0.62-fold, 0.41-fold and 0.34-fold after replacing fish oil with soybean oil at up to 60%, 80%, and 100%, respectively (*p* < 0.05). In addition, the expression levels of *Δ9 fad* did not differ significantly across the SO-0, SO-20 and SO-40 treatments (*p* > 0.05) (Figure 5).

### 3.5. Transcriptional Levels of Δ9 fad in Response to Chronic Low-Salinity Stress

**Figure 6:**
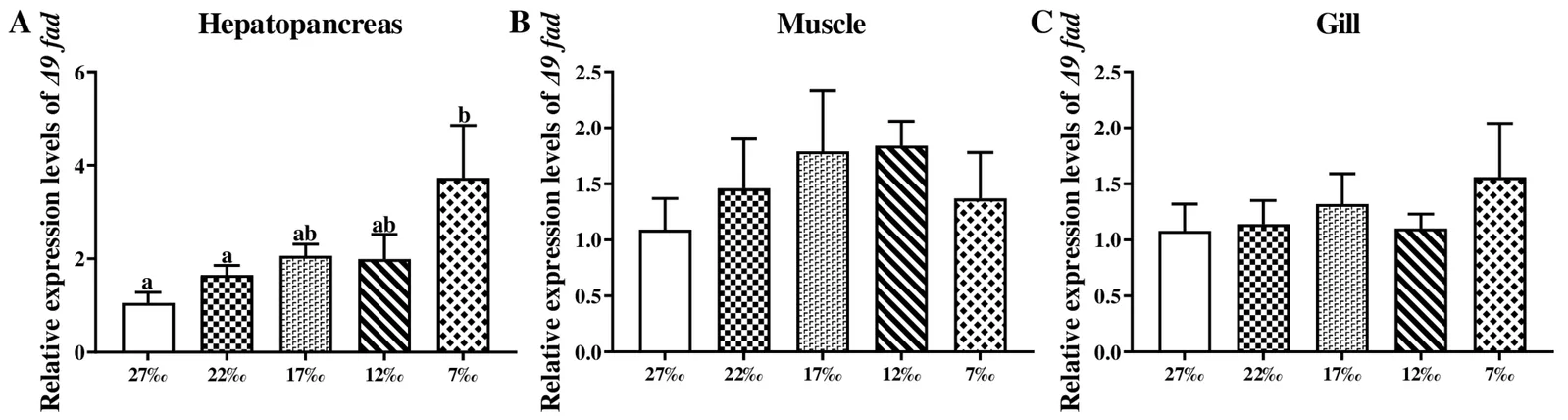
Transcriptional responses of *Scylla paramamosain* Δ9 fatty acyl desaturase in hepatopancreas (**A**), muscle (**B**) and gill (**C**), under chronic salinity challenge. Values are expressed as mean ± standard error of mean (SEM, *n* = 6). Different superscript letters denote significant differences (*p* < 0.05). Numerals correspond to seawater salinity.

The results showed that the transcriptional abundance of *Δ9 fad* and *srebp-1* in the hepatopancreas was significantly higher in the 7‰, 12‰, 17‰, and 22‰ salinity groups compared with the 27‰ group, with a significant difference detected between the 7‰ and 27‰ salinity treatments (*p* < 0.05). In muscle, no remarkable intergroup variations were observed in the transcript levels of *Δ9 fad* and *srebp-1*, even though the 27‰ salinity group showed lower values than the 7‰, 12‰, 17‰, and 22‰ salinity groups (*p* > 0.05). Moreover, salinity treatments exerted no obvious effects on *Δ9 fad* expression in the gill (*p* > 0.05) (Figure 6 and Figure S1).

### 3.6. Transcriptional Levels of Δ9 fad in Response to Acute Low-Salinity Stress

**Figure 7:**
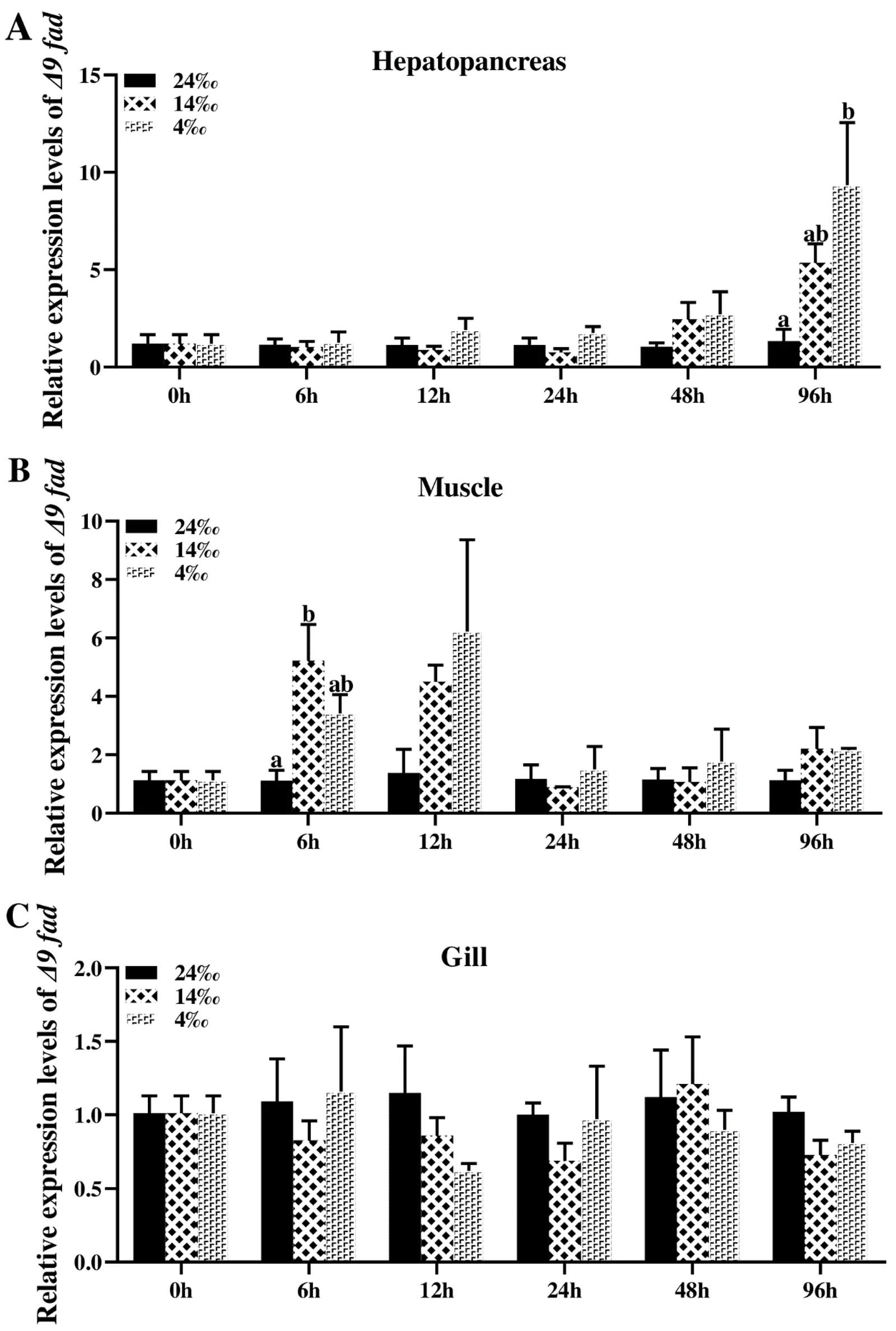
Transcriptional responses of *Scylla paramamosain* Δ9 fatty acyl desaturase in gill, muscle and hepatopancreas under acute salinity challenge. Total RNA was extracted from hepatopancreas (**A**), muscle (**B**), and gill (**C**) at serial time points following exposure to 24‰, 14‰ and 4‰ salinity treatments, respectively. Crabs maintained at 24‰ served as control. Statistical comparisons were performed between each treatment group and control group at identical sampling time points. Different superscript letters denote significant differences (*p* < 0.05).

To explore the transcriptional responses of *Δ9 fad* to acute low-salinity challenge, total RNA was extracted from the gill, muscle and hepatopancreas at multiple sampling time points. The effects of acute low-salinity challenge on *Δ9 fad* transcript abundance in the gill, muscle and hepatopancreas are shown in Figure 7. In comparison with the 24‰ salinity control group, hepatopancreatic *Δ9 fad* transcription was upregulated in the 4‰ salinity groups at 96 h, 48 h, 24 h, 12 h and 6 h, while only the 4‰ salinity group at 96 h exhibited remarkably higher mRNA levels relative to the 24‰ salinity group (*p* < 0.05). In muscle, the transcriptional levels of *Δ9 fad* in the 14‰ and 4‰ salinity groups showed an obvious increasing trend compared with the 24‰ salinity group at 12 h and 6 h. In addition, there were no significant differences in the *Δ9 fad* expression in the gill among the 4‰, 14‰ and 24‰ salinity treatments across all sampling time points (Figure 7).

### 3.7. Transcriptional Levels of Δ9 fad in Response to Starvation Stress

**Figure 8:**
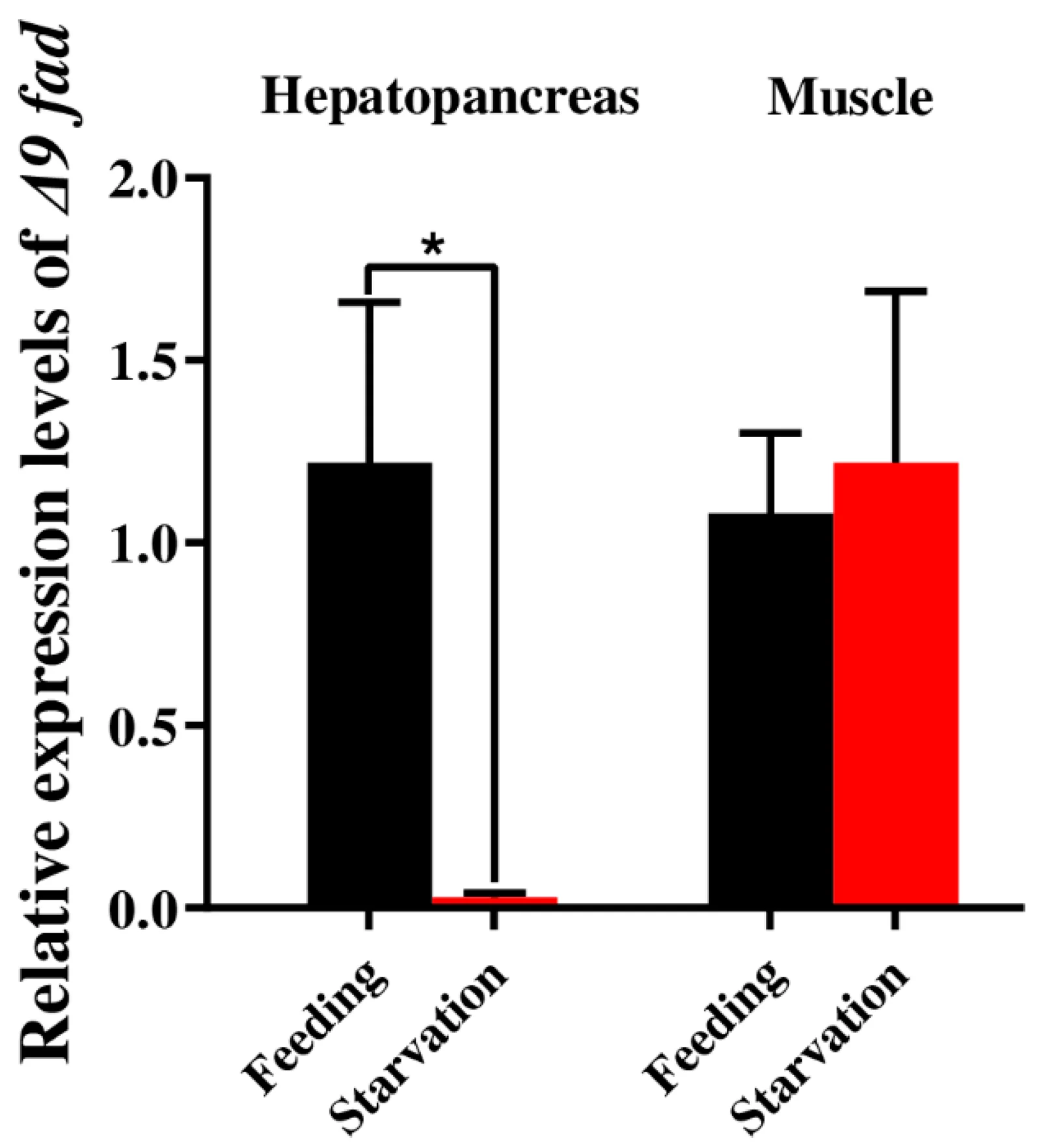
Transcriptional responses of *Scylla paramamosain* Δ9 fatty acyl desaturase in muscle and hepatopancreas under starvation stress. Asterisks denote significant difference between starvation group and feeding group (*p* < 0.05).

Compared with the feeding group, the transcriptional levels of hepatopancreatic *srebp-1* and *Δ9 fad* were significantly downregulated in the starvation group (*p* < 0.05). By contrast, the mRNA expression of *Δ9 fad* and *srebp-1* in muscle exhibited no significant difference between the two groups (*p* > 0.05) (Figure 8 and Figure S2).

## 4. Discussion

Δ9 FAD functions as a lipogenic enzyme and exerts pivotal effects on MUFA synthesis, and these fatty acids are vital constituents of triglycerides, wax esters and membrane phospholipids [2,3]. In this study, full-length *Δ9 fad* cDNA was successfully isolated from the hepatopancreas of mud crab. The Δ9 FAD-like domain of mud crab encodes a 347-amino-acid protein, which shares high homology with previously reported Δ9 FADs from other crustaceans like *E. sinensis* (83%), *G. lalandii* (86%), *P. trituberculatus* (96%) and *S. olivacea* (98%), indicating that these genes are evolutionary conserved. Sequence analysis indicated that the putative protein possesses the typical signatures of Δ9 FAD, consisting of four transmembrane regions, three conserved motifs (FFSHMGW, QRKYY and PWDY) and three histidine-rich domains (HX_4_H, HX_2_HH and HX_2_HH), which was similar to the structural characteristics of Δ9 FAD from other species [3,4,8,9]. The eight conserved histidine residues serve as non-heme iron-binding sites and are indispensable for Δ9 FAD catalytic activity [3,5]. In addition, phylogenetic analysis showed that mud crab Δ9 FAD grouped with crustacean orthologs and formed a tight cluster with Δ9 FAD from *S. olivacea* and *P. trituberculatus*, indicating a close genetic relationship among them. Collectively, the results from homology, structural characteristics and phylogenetic analysis support that the desaturase identified in this study belongs to Δ9 FAD.

Stearoyl-CoA desaturase (SCD, also known as Δ9 FAD) widely exists in higher organisms, and mammalian species display considerable variability in the gene complement [4]. So far, four mouse *scd* isoforms (1–4) and two human *scd* isoforms (1 and 5) have been recognized, and these genes exhibit tissue-dependent expression patterns corresponding to the tissue-specific functions in fatty acid metabolism [2]. Similarly, *Δ9 fad* exhibits different expression patterns in distinct tissues of various aquatic animals. Studies in *Larimichthys crocea* [20], *Epinephelus coioides* [11] and *Ctenopharyngodon idella* [28] have shown that *scd-1* was principally expressed in the liver, followed by the brain. By contrast, *scd-1* was expressed across all tested tissues in *Dicentrarchus labrax*, and expression abundance was higher in adipose tissue than in the gonad, liver, intestine and heart [29]. In addition, the brain and liver exhibited far higher *scd-1* mRNA levels in *Pampus argenteus*, with the intestine and kidney showing comparatively lower expression [14]. In this study, only a single *Δ9 fad* homolog was obtained from the mud crab genome and transcriptome resources, without detectable paralogs, and *Δ9 fad* was highly expressed in the hepatopancreas of mud crab relative to other tissues. This finding agrees with the results documented in *E. sinensis* [8] and *C. quadricarinatus* [9], in which *Δ9 fad* is predominantly expressed in the hepatopancreas. The higher expression of *Δ9 fad* in the hepatopancreas is consistent with its functions in lipid metabolism and storage, similarly to the insect fat body and vertebrate liver [30]. Moreover, prominent *Δ9 fad* transcript levels were observed in cranial ganglia and thoracic ganglia in this study, which may indicate that *Δ9 fad* plays a key role in these tissues of mud crab.

Previous studies have indicated that *Δ9 fad* expression can be significantly modulated by dietary fatty acids [3,4]. The mice fed an SFA-rich diet showed markedly higher hepatic *scd-1* transcriptional levels than those fed a normal diet, and compared to linseed or fish oil, beef tallow (rich in SFAs) significantly upregulated SCD-1 activity in the liver of rats [4,31]. Oleic acid (C18:1n-9), representative of MUFAs, was applied to explore lipid metabolism-related gene expression. The results demonstrated that C18:1n-9 did not affect the expression of *srebp-1c* or fatty acid synthetase (*fas*), and accordingly, it can be speculated that *scd-1* expression would barely change [4]. In addition, studies showed that diets supplemented with PUFAs like arachidonic acid (C20:4n-6), linolenic acid (C18:3n-3) and linoleic acid (C18:2n-6) can observably repress *scd-1* expression through inhibiting transcription factors like *srebp-1c* [3]. In the present study, the results exhibited that soybean oil substitution for fish oil markedly downregulated *Δ9 fad* expression in the hepatopancreas of mud crab. This finding is consistent with a prior investigation on *E. sinensis* [8], in which soybean oil-containing diet elicited a significant reduction in *Δ9 fad* mRNA abundance in the hepatopancreas relative to the fish oil diet. On the contrary, Lochsen et al. [32] observed higher hepatic *scd-1* mRNA levels in rats fed with soybean oil relative to those fed with fish oil. The above findings may indicate that the precise mechanisms regulating *Δ9 fad* expression are complex, encompassing species-specific regulatory patterns of SREBP-1, variations in the relative proportions of C18:2n-6 and C18:1n-9 in experimental diets, and divergent tissue-specific metabolic responses. In this study, the soybean oil used in experimental diets possesses higher C18:1n-9 content than fish oil, and C18:1n-9 is the product generated by Δ9 FAD. Thus, one possible explanation for the present result is that high concentrations of C18:1n-9 may inhibit *Δ9 fad* expression to repress fatty acid synthesis.

In addition, *Δ9 fad* expression can also be markedly influenced by environmental factors [9,18,19,20,21,33]. Previous studies have demonstrated that Δ9 FAD performs a critical function in adaptation to cold stress for poikilothermic animals through elevating phosphoglyceride unsaturation to recover the fluidity of membranes stiffened under low temperature [4]. Hsieh and Kuo indicated that the enzyme activity of Δ9 FAD was closely linked to the tolerance of *Ctenopharyngodon idella* and *Chanos chanos* to low temperature. In *L. crocea*, the expression levels of scd-1 in the liver and brain were markedly upregulated when the water temperature dropped from 15 °C to 7 °C [20]. A similar result was also reported in *C. quadricarinatus*, in which it was found that compared with the 25 °C water temperature groups, the *Δ9 fad* mRNA levels in the hepatopancreas were significantly increased in the 9 °C, 15 °C and 20 °C groups, and the 9 °C water temperature group exhibited the highest value [9]. Excluding water temperature variation, salinity and starvation are also common stress factors, and the present study determined the roles of *Δ9 fad* in coping with salinity and starvation. The results from chronic salinity challenge indicated that the low-salinity groups exhibited higher *Δ9 fad* mRNA levels in the hepatopancreas of mud crab than the normal seawater group (27‰), especially in the 7‰ salinity groups, showing that chronic salinity stress can stimulate *Δ9 fad* expression, possibly because low salinity induced the expression of transcription factor *srebp-1* and then further facilitated *Δ9 fad* expression (downstream target gene) for MUFA synthesis to maintain membrane fluidity. In addition, the *Δ9 fad* transcriptional levels of the muscle and gill were unaffected by chronic salinity stress, implying that the hepatopancreas plays a predominant role in mediating responses to chronic salinity stress. Under acute salinity stress, the muscle, as an important osmoreceptor, also participates actively in the response to low-salinity stress. The *Δ9 fad* transcriptional levels of muscle in the 14‰ and 4‰ salinity groups were higher than those in the 24‰ salinity group at 6 h and 12 h. In contrast, hepatopancreatic *Δ9 fad* exhibited a delayed response to low-salinity stress, and only the 4‰ salinity group at 96 h had a higher value than the 24‰ salinity group. These results indicate that the muscle exhibits greater sensitivity to low-salinity stress relative to the hepatopancreas, whereas the hepatopancreas serves a critical function in coping with prolonged salinity stress. Consistently, An et al. [33] reported that compared with the 32‰ salinity group (normal seawater), the *Δ9 fad* expression of *Chlamydomonas* was markedly elevated in the 24‰ and 16‰ salinity groups. Accordingly, C18:1n-9, the product catalyzed by Δ9 FAD, exhibited significantly higher levels in these two low-salinity groups compared with the 32‰ salinity group. These findings demonstrate the involvement of *Δ9 fad* in salinity adaptation, further corroborating the observations obtained in the present study. With regard to the starvation stress trial, the transcriptional levels **of** *Δ9 fad* in the hepatopancreas were dramatically reduced in the starvation group relative to the feeding group. A possible explanation is that lipids (mainly triglycerides) are more prone to oxidation for providing supply energy rather than de novo synthesis. Consequently, *Δ9 fad*, which encodes a lipogenic enzyme, was strongly suppressed under starvation conditions in this study.

## 5. Conclusions

In summary, the complete cDNA sequence of *Δ9 fad* was obtained from the hepatopancreas of mud crab using RACE technology. Homology analysis, structural characterization and phylogenetic analysis collectively confirmed that the desaturase identified herein corresponds to Δ9 FAD. Tissue expression profiling revealed that *Δ9 fad* exhibited the highest transcript abundance in the hepatopancreas, implying its predominant function within this tissue. Furthermore, this study reveals a clear tissue-specific function of *Δ9 fad* in coordinating lipid metabolism and stress adaptation. The hepatopancreas serves as the primary long-term metabolic regulator, where Δ9 fad sustains stable fatty acid desaturation and continuous lipid metabolic homeostasis to support routine physiological maintenance. In contrast, muscle functions as a sensitive acute stress sensor, in which Δ9 fad rapidly remodels metabolic profiles to cope with short-term stress. These findings advance our understanding of the functions of *Δ9 fad* and its underlying regulatory mechanisms in crustaceans. Nevertheless, the present study relies solely on gene expression data without measurements of enzymatic activity and fatty acid composition, necessitating future research integrating enzyme activity assays and fatty acid profiles to further validate these findings.

## Figures and Tables

**Table 1:** Primer names and sequences employed in this study.

Primer	Sequence (5′-3′)	Objective
Oligo-	AAGCAGTGGTATCAACGCAGAGTACXXXXX	First-strand cDNA synthesis
UPM (long)	CTAATACGACTCACTATAGGGCAAGCAGTGGTATCAACGCAGAGT	RACE-PCR
UPM (short)	CTAATACGACTCACTATAGGGC	RACE-PCR
NUP	AAGCAGTGGTATCAACGCAGAGT	RACE-PCR
Δ9 Fad 3-1	ACGACTTGCTAGAGGGTGAAGAGGA	3′RACE
Δ9 Fad 3-2	GTCCTATGTTCCAGTGTACGCCAAG	3′RACE
Δ9 Fad 5-1	CGTCTGTCTCGCTGTACTTGTGAT	5′RACE
Δ9 Fad 5-2	CAGATTTCGCAGGTCGGAGAG	5′RACE
C-Δ9 Fad F	CCGCCATTCATTCAGTGC	Verify sequence
C-Δ9 Fad R	GTTGCGTGGTGGGTCGT	Verify sequence
Q-Δ9 Fad F	TGGTGGCTTACCTGTCCCTG	qRT-PCR
Q-Δ9 Fad R	GCGTCTGTCGATCATTTCCG	qRT-PCR
β-actin R	GCGGCAGTGGTCATCTCCT	qRT-PCR
β-actin F	GCCCTTCCTCACGCTATCCT	qRT-PCR
M13F	CGCCAGGGTTTTCCCAGTCACGAC	PCR screening
M13R	AGCGGATAACAATTTCACACAGGA	PCR screening

X: undisclosed base in the proprietary SMARTer oligo sequence.

**Table 2:** Sequence identities of *Scylla paramamosain* Δ9 fatty acyl desaturase with other crustaceans.

Matched Species	GenBank Accession Number	% Identity
*Scylla olivacea*	QKG32713	98
*Portunus trituberculatus*	XP_045124725	96
*Gecarcoidea lalandii*	QKG32710	86
*Eriocheir sinensis*	XP_050736646	83
*Penaeus japonicus*	XP_042859426	80
*Penaeus monodon*	XP_037782903	80
*Penaeus chinensis*	XP_047496645	80
*Cherax quadricarinatus*	XP_053644453	78
*Macrobrachium nipponense*	AMQ48727	76
*Procambarus clarkii*	XP_045609654	68

## Data Availability

Data are available from the corresponding author upon reasonable request. The *Δ9 fad* sequence was deposited in GenBank under the accession number MF124319 (https://www.ncbi.nlm.nih.gov/, accessed on 17 August 2026).

## Supplementary Materials

The following supporting information can be downloaded at https://www.mdpi.com/article/10.3390/life16081372/s1, Figure S1: Transcriptional responses of *Scylla paramamosain* sterol-regulatory element-binding protein-1 (*srebp-1*) in hepatopancreas and muscle under chronic low-salinity challenge; Figure S2: Transcriptional responses of *Scylla paramamosain* sterol-regulatory element-binding protein-1 (*srebp-1*) in muscle and hepatopancreas under starvation stress. Table S1: Ingredient formulation and proximate nutritional profiles of experimental diets (dry matter basis, g kg^−1^); Table S2: Fatty acid profiles of experimental diets (expressed as % total fatty acids).

## Abbreviations

The following abbreviations are used in this manuscript:

Δ9 FAD	Δ9 fatty acyl desaturase
MUFA	Monounsaturated fatty acid
CHREB	Carbohydrate response element-binding protein
LXR	Liver X receptor
C18:2n-6	Linoleic acid
C18:3n-3	Linolenic acid
C20:5n-3	Eicosapentaenoic acid
