# Supplementary material for: Molecular Cloning and Functional Characterization of Δ9 Fatty Acyl Desaturase in Scylla paramamosain: Insights into Its Regulatory Roles Under Salinity Challenge, Dietary Fatty Acid Alteration and Starvation Stress
Source: Life (Basel). 2026 Aug 20;16(8):1372. doi: 10.3390/life16081372 (PMC13514243; doi:10.3390/life16081372)
Supplement: Supplementary file 1 [file life-16-01372-s001.zip › life-4463235-supplementary.pdf]

Table S1. Ingredient formulation and proximate nutritional profiles of the experimental diets (dry matter basis, g kg^{-1})

	Diets [1]					
	SO0	SO20	SO40	SO60	SO80	SO100
Ingredient composition						
Fish meal [2]	410	410	410	410	410	410
Casein [3]	180	180	180	180	180	180
Fish oil [4]	80	64	48	32	16	0
Soybean oil [5]	0	16	32	48	64	80
Dextrin	220	220	220	220	220	220
Vitamin mix [6]	30	30	30	30	30	30
Mineral mix [6]	20	20	20	20	20	20
Cholesterol	8	8	8	8	8	8
Lecithin	10	10	10	10	10	10
Monocalcium phosphate	15	15	15	15	15	15
Choline chloride	7	7	7	7	7	7
Sodium alga acid	10	10	10	10	10	10
Squid liver paste	10	10	10	10	10	10
Proximate composition (g kg^{-1})						
Moisture	99	98	103	102	104	104
Crude protein	457	455	458	455	453	449
Crude lipid	95	99	94	101	96	97
Ash	110	109	104	104	106	108

[1] SO0 means that fish oil is the only dietary lipid source, numerical values after SO refer to the percentage of dietary fish oil replaced by soybean oil.

[2] Supplied by Coastal Villages Pollock, Anchorage, AK, USA.

[3] Supplied by Hualing Casein Co. Ltd., Gansu, China.

[4] Supplied by Improved Variety Introduction Co. Ltd., Guangdong, China.

[5] Supplied by Shenzhen Nanshun Lipid Co. Ltd., Guangdong, China.

[6] Supplied by Xingmuwei Animal Health Product, Xiamen, Fujian, China.

Table S2. Fatty acid profiles of experimental diets (expressed as % total fatty acids)

Fatty acid [1]	Diets [2]					
	SO0	SO20	SO40	SO60	SO80	SO100
14:0	3.2	2.6	2.2	1.8	1.4	0.6
16:0	15.2	14.5	13.8	13.3	12.1	11.6
18:0	4.4	4.5	4.7	4.8	4.9	4.9
20:0	1.4	1.3	1.1	0.9	0.8	0.7
22:0	0.7	0.6	0.5	0.5	0.3	0.2
16:1n-7	3.9	3.2	2.8	2.2	1.3	0.7
18:1n-9	19.2	22.3	24.1	26.2	28.4	29.7
20:1n-9	1.5	1.3	1.2	1.2	1.2	1.1
22:1n-11	0.4	0.4	0.3	0.2	0.2	0.1
18:2n-6	19.1	25.0	30.2	34.5	37.6	41.6
18:3n-6	1.7	1.4	1.0	0.8	0.6	0.4
18:3n-3	3.0	3.3	3.8	4.1	4.5	4.9
20:2n-6	0.9	0.8	0.5	0.4	0.4	0.2
20:3n-3	3.2	2.5	2.0	1.5	0.9	0.5
20:4n-6	1.0	0.8	0.7	0.6	0.3	0.1
20:5n-3	9.1	6.7	4.5	3.3	2.3	1.1
22:6n-3	11.9	8.8	6.4	3.7	2.8	1.5
ΣSFA [3]	24.9	23.5	22.3	21.3	19.5	18
ΣMUFA [4]	25.0	27.2	28.4	29.8	31.1	31.6
ΣPUFA [5]	24.7	30.5	35.5	39.8	43.1	47.1
ΣHUFA [6]	25.2	18.8	13.6	9.1	6.3	3.2
Σn-3 FA [7]	27.2	21.3	16.7	12.6	10.5	8
Σn-6 FA [8]	22.7	28.0	32.4	36.3	38.9	42.3
n-3/n-6	1.2	0.8	0.5	0.3	0.3	0.2

[1] Data expressed as area percentage of fatty acid methyl ester, represent mean ± SEM, n = 3. Fatty acids present at ≤ 0.1 percentage of total fatty acids are not included.

[2] Numerical values after SO refer to the percentage of dietary fish oil replaced by soybean oil.

[3] SFA: 14:0, 16:0, 18:0, 20:0, 22:0.

[4] MUFA: 16:1n-7, 18:1n-9, 20:1n-9, 22:1n-11.

[5] PUFA: 18:2n-6, 18:3n-6, 18:3n-3, 20:2n-6.

[6] HUFA: 20:3n-3, 20:4n-6, 20:5n-3, 22:5n-3, 22:6n-3.

[7] n-3 FA: 18:3n-3, 20:3n-3, 20:5n-3, 22:6n-3.

[8] n-6 FA: 18:2n-6, 18:3n-6, 20:2n-6, 20:4n-6.

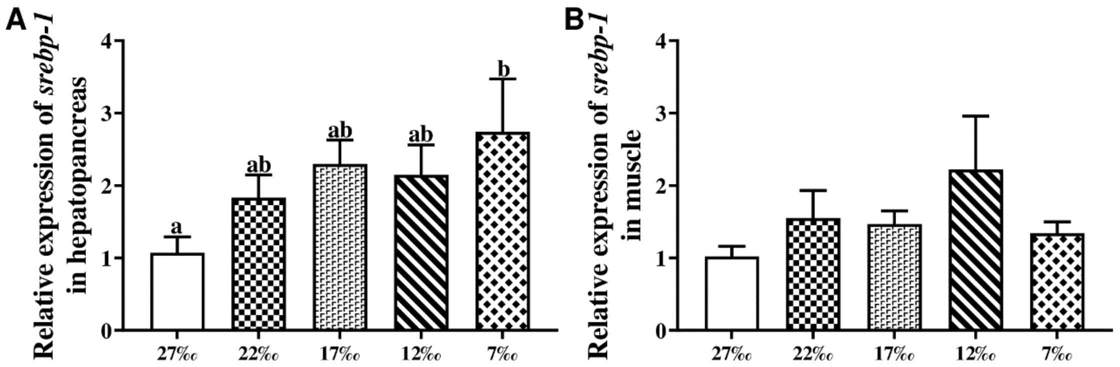

Figure S1. Transcriptional responses of *Scylla paramamosain* sterol regulatory element binding protein-1 (*srebp-1*) in hepatopancreas and muscle under chronic low salinity challenge. Values are expressed as mean ± standard error of the mean (SEM, n = 6). Different superscript letters denote significant differences ($p < 0.05$). Numerals correspond to seawater salinity.

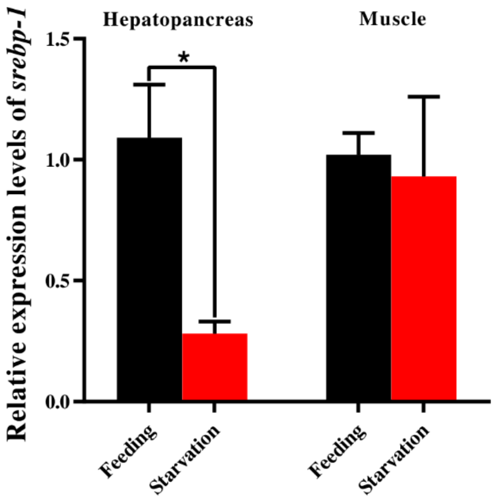

Figure S2. Transcriptional responses of *Scylla paramamosain* sterol regulatory element binding protein-1 (*srebp-1*) in muscle and hepatopancreas under starvation stress. Asterisks denote significant difference between starvation group and feeding group ($p < 0.05$).
